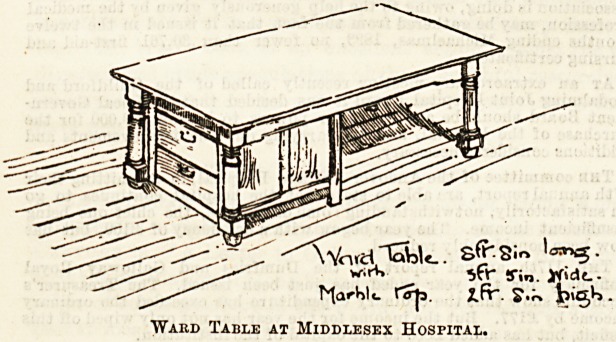# Ward Fittings at Middlesex Hospital

**Published:** 1894-02-10

**Authors:** 


### PRACTICAL DEPARTMENTS.

### WARD FITTINGS AT MIDDLESEX HOSPITAL.

The cheerful, well-fitted wards of Middlesex Hospital are well worth a visit from all who want to see for themselves what a large measure of comfort and care is provided for patients within the walls of a modern hospital. As our readers know, Middlesex Hospital has recently undergone immense improvements, and modern appliances of all kinds are now to be found there.

In the Murray Ward, which is a women's medical ward, many comforts are provided. Wheel chairs and cosy couches are there, and a new idea in the way of ward benches seemed so excellent that we obtained the kind permission of the Matron, Miss Thorold, to reproduce a sketch, which will clearly show its special advantages.

WARD BENCH AT MIDDLESEX HOSPITAL.

The table and cupboard combined, which forms the subject of the second illustration, is placed in the centre of the wide ward, and the benches are ranged on either side, though they are, of course, perfectly easy to move if desired. The special point which the drawing brings out is the moveable back, made, as will be seen, to hinge, so that patients can seat themselves either side, thus obviating the necessity of moving the whole bench according to whether the patients wish to set up to the table or to face the opposite way.

They are of a comfortable height and very well made and finished, the hinged back acting with ease. The dimensions are given in the illustration. Both benches and tables are made in polished wood.

WARD TABLE AT MIDDLESEX HOSPITAL.

The table, one end of which is fitted with convenient cupboards and drawers, is marble topped, and well-proportioned in size. It is sufficiently low to make a very comfortable dining-table for convalescent patients, and when not thus in use is quite an ornament to the ward with its palms and ferns in bright-coloured pots.

Both table and bench here described are each excellent in their several ways and of much practical use. The little points in their construction which show consideration for the comfort of the sick people for whose use they are intended, are small in themselves, but make just all the difference in degrees of comfort, and we heartily recommend their adoption in other hospital wards.

## Figures and Tables

**Figure f1:**
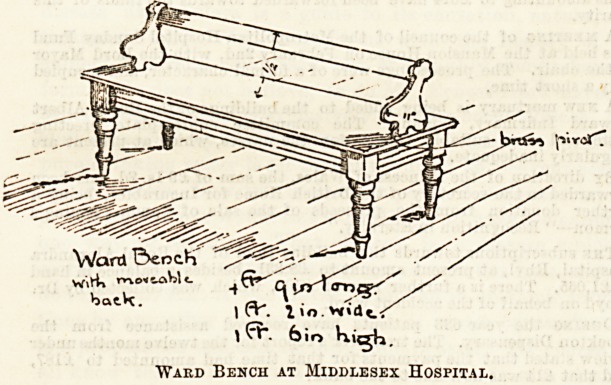


**Figure f2:**